# Modern Views on Heredity

**Published:** 1909-04-17

**Authors:** R. Murray Leslie

**Affiliations:** Senior Physician to the Prince of Wales's. General Hospital, Physician to the Royal Hospital for Diseases of the Chest


### Hospital Clinics.

### MODERN VIEWS ON HEREDITY.

By R. MURRAY LESLIE, M.A., B.Sc., M.D., M.R.C.P.; Senior Physician to the Prince of Wales's General Hospital, Physician to the Royal Hospital for Diseases of the Chest.

(A Lecture Delivered at the N.E. London Post-Graduate College.)

Heredity is a general term signifying the genetic continuity which links one generation with another. It implies, as Galton well expresses it, that "like tends to produce like." The physical basis of heredity is the fertilised ovum, which, according to Darwin, is "the most wonderful object in nature." It is, indeed, difficult to comprehend the extraordinary molecular complexity and physiological potentialities that reside in the ovum and spermatozoon. The reproductive cell of a woman measures only $\frac{1}{120}$ inch, while the spermatozoon is only $\frac{1}{100000}$ of the size of the ovum. And yet the potentiality of this minute speck of protoplasm is so marvellous that a son not only resembles his father in external appearance, but reproduces his very gestures, and, it may be, the tones of his voice.

Weismann regards the chromatin network of the nucleus of the germ cell as the bearer of the hereditary characters. It would serve no useful purpose for me to go into the question of the extrusion of polar cells preceding the ultimate fusion of the male and female germ cells. We must, however, briefly refer to the principal theories of inheritance and the laws which govern its manifestations. We will also touch upon some of the more interesting problems of heredity.

Darwin's theory of *pangenesis* suggests that all the cells of the body give off representative gemmules, which find their way somehow to the reproductive elements, so that the latter come to contain representative samples of the various component parts of the body, and are, therefore, able to develop into an offspring like the parent. This theory has been largely discarded.

Weismann's theory of "*germinal continuity*" or "continuity of the germ plasm" is the hypothesis which has obtained most adherents. Weismann holds that when the parent's body is developing from the fertilised ovum, a residue of unaltered germinal material is kept apart to form the future reproductive cells, one of which may become the starting-point of a child. The germinal material which starts an offspring is thus maternally continuous with the germinal material from which the parent arose.

Professor Francis Darwin, in his address to the British Association last year, set forth some of the difficulties which exist in accepting either his distinguished father's theory of pangenesis or Weismann's theory of germinal continuity as affording a satisfactory explanation of heredity. He enunciated a new theory which he called the "*mnemic*" (memory) theory, based on the conjecture that stimuli are not necessarily ephemeral, but leave a trace or record—"*engram*"—on the organism. In dealing with the movements of plants, Professor Darwin showed how changes in environment act as stimuli, and this is true not only of temporary changes of shape, but also of permanent alterations. He suggested that the dim beginnings of habit or unconscious memory in the movements of plants and animals must find a place in morphology. If external stimuli may thus determine, not only the first stages in development, but also elaborate structural differences, we must then allow, with whatever words we disguise the admission, the inheritance of acquired characters.

### Bi-parental Heredity.

In the higher forms of life, parthenogenesis must be regarded as extremely exceptional, though it is common enough in some of the lower forms. For all we know to the contrary it may occur in an infinitesmal number of cases even in the human species.

At present we need only deal with sexual reproduction. From both parents comes the inherited organisation, which, according to Weismann, has its seat in the chromosomes of the nuclei of the ovum and spermatozoon. From the father comes the centrozome which organises the system of cell-cleavage and distributes the dual inheritance between the daughter cells. Huxley, as far back as 1878, stated that it is conceivable, and indeed probable, that every part of the adult contains molecules derived both from the male and female parent, and that, regarded as a mass of molecules, the entire organism may be compared to a web, of which the warp is derived from the female and the woof from the male. There is thus, as Professor Arthur Thomson expresses it, an intimate and orderly union of maternal and paternal elements.

The characters of both parents may be "blended" in the offspring, or the resemblance may be "unilateral" (the child resembling one parent) and often crossed, the son taking after the mother and the daughter after the father. In respect of certain characters the paternal inheritance is often more potent than the maternal, and *vice versa*. Weismann made a theoretical suggestion that there may be a germinal struggle in the arcana of the germ cells, a struggle in which the maternal and paternal contributions may blend and harmonise, or may neutralise one another, or in which one may conquer the other, or in which both may persist without combining.

The fact that a particular character—such as a diseased condition—sometimes skips a generation simply implies that in the intermediate generation the predisposition was there latent and unexpressed owing to the absence of the appropriate stimulus. Thus in the case of hereditary insanity, if a girl in one generation makes a happy love marriage she may remain normal throughout life, while her daughter, who may suffer a severe love disappointment, may get an attack of acute mania.

### Law of Filial Regression towards Mediocrity.

There is a tendency for children of exceptional parents to regress towards the average stock. Galton terms this tendency filial regression. This applies equally to exceptional physical and mental characters. Thus, though tall stature may run in certain families, yet there is always a tendency to revert to the mean average size. Similarly, the children of a genius tend to have somewhat less than their father's power, but more than the average of the race. According to Professor Pearson, distinguished parents are just ten times more likely to have distinguished offspring than undistinguished parents. Still such cases as the Darwins, father and sons, the two Pitts, Philip and Alexander the Great are exceptional. Similarly, also the children of a criminal tend to be less vicious than the father, though morally inferior to the average man.

### The Law of Ancestral Inheritance.

Galton's studies in connection with Basset Hounds enabled him to formulate a most important law in regard to inheritance. According to this *the two parents between them contribute on an average one half of each inherited faculty, each of them contributing one quarter; the four grandparents contribute between them one quarter, each contributing one sixteenth, and so back ad infinitum.*

*Reversion*, according to Professor Pearson, signifies the reappearance in one individual of a character which is recorded to have occurred in a definite ancestor of the same race; while *Atavism* signifies a similar reappearance of a character, not typical of the race, but found in allied races, supposed to be related to the evolutionary ancestry of the given race. Most authorities use the two terms as synonymous. Reversion simply means that certain potentialities, which form part of the heritage, may remain dormant and unexpressed for lack of the appropriate liberating stimulus.

Mendel, in connection with his studies in the crossing of plants, made a discovery that in cases where dissimilar characters meet in one individual there is, on the formation of germ cells, a separation—" segregation "—between the two characters. One of these characters is spoken of as the " dominant " and the other the " recessive "; in the case of each pair of characters the " dominant " is the one which in the first cross prevails to the exclusion of the other—the " recessive." The numerical proportion of dominants to recessives in the first cross is, on an average, 3 to 1. In the first generation, therefore, derived from the cross, there will be 75 per cent. of " dominants " and 25 per cent. of " recessives." The offspring of the recessives remain pure recessive, and in subsequent generations never produce the dominant again; they thus breed true. In the case of dominants, however, two classes of offspring result, one consisting of pure dominants and the other of mixed offspring composed partly of recessives and partly of dominants. The normal proportion of pure dominants to mixed offspring being as 1 to 2. The pure dominants, like the pure recessives, breed true, and only give rise to dominants in succeeding generations. To take an illustration; in the case of eye colour in men, the presence of pigment is a dominant, while in the case of the malady known as colour-blindness, the blindness is dominant in males and recessive in females, according to Professor Bateson. This observer states that a large number of hereditary diseases follow Mendel's " Law of Heredity."

*Telegony* is a term which has been applied to those cases where an offspring resembles not so much its own father, but a previous mate of its mother. From the evidence collected by Romanes and others there seems to be little doubt—in spite of much disbelief—that the male may, through effective impregnation of a female, exert such an influence on the mother that the subsequent progeny born by the same female to a different sire resemble the father of the first-born. It is indeed an axiom with all breeders of thoroughbred stock that to keep a strain pure there must be no admixture with stock of another blood. Thus in the case of sporting dogs, if a female has been accidentally crossed by a collie, her value as a mother of pure-bred dogs becomes greatly diminished, as in the future, even though mated with a pure-bred sire, she may not breed true, but may bear progeny partaking of the collie character. The most notable instance is the oft-quoted case of Lord Morton's Arabian mare, which was experimentally mated with a quagga, and produced a foal marked with zebra-like stripes. Subsequently the same mare had two other foals sired by an Arab horse, and these also showed less distinct zebra-like markings.

Sir William Turner, discussing this case, expressed his belief that the mother had acquired during her prolonged gestation of the hybrid, the power of transmitting quagga-like characters owing to the interchange of material, nutritive or otherwise, which had taken place in connection with the nutrition of the embryo. In this way the germ-plasm of the mother belonging to ova which had not yet matured had become modified while still lodged in her ovary. The theory of telegony, if correct, illustrates how the germ-plasm may be directly influenced by the soma, and would also serve to explain the transmission of acquired somatogenic characters.

Is there any ground for believing that a woman's first husband, who perchance may have had an hereditary tendency to some disease, may so impress his wife that after his death she may transmit the hereditary taint to her future children by a healthy husband? It is only right to mention that many of these so-called cases of telegony are believed by many competent authorities to be simply cases of reversion to some remote ancestor, though it seems impossible to explain all cases on such an hypothesis. Professor Arthur Thomson believes that some physiological influence, as yet not understood, may pass from the male to the female distinct from the act of conception.

### Theory of Maternal Impression.

This is a subject of great interest, the belief in which has prevailed in all nations from the earliest times. Even at the present time most women are convinced that emotions and mental states during pregnancy may so affect the fœtus as to impress upon it definite structural alterations. Biblical students will readily recall Jacob's hebraic foresight in adding to his flocks and herds by setting peeled straked rods before them, so that the sheep and kine straightway brought forth ring-straked, speckled and spotted offspring. Personally I have no hesitation in affirming my belief in the possible transmission of psychological characteristics from the pregnant woman to the developing embryo *in utero*. It is, indeed, conceivable that such influences passing from women endowed with refinement, culture, and high aspirations may have far-reaching effects in promoting the higher evolution of the species. . . . . The mother of the future may recognise her responsibility in regard to the results which her life and conduct may have on the welfare of her unborn child. Last year I showed at the North Eastern Clinical Society a ten-months-old child, whose back was completely covered by a large brown hairy nævus, the occurrence of which was attributed by the mother to her having had during her pregnancy a severe shock from witnessing a fight between two dogs, one of which was brown.

Dr. Ballantyne, the distinguished teratologist, records an instance of a woman who, when three months pregnant, saw one of her children nearly amputate the middle, ring, and little fingers of his left hand by playing with a chopper. When her child was born it was found to be minus the middle, ring, and little fingers of the left hand. It is difficult to explain such a case by the theory of mere coincidence. Personally, I think there is little doubt that emotional or mental states of the mother may in different ways affect the development of the fœtus *in utero*. Many of the foremost obstetricians in America are strong believers in the potency of maternal impression, while other authorities equally eminent, emphatically express their disbelief in the doctrine, the validity of which must therefore remain *sub judice*.

### Origin of Variations.

Little is known in regard to the origins of variations, but they are generally supposed to result from changes in the germinal matter, either before or during fertilisation. Weismann in alluding to the discovery that each ovum and spermatozoon loses half its chromosomes, and that the fertilised ovum thus contains only a portion of the elements of each, explains variation by suggesting that there is a germinal selection of parental characteristics, and that therefore the new individual may vary from either of his parents. Professor Pearson, on the other hand, holds that variability is not in any sense a product of sexual or bi-parental inheritance; while Archdale Reid goes further, and brings forward evidence to prove that bi-parental reproduction, instead of predisposing to variability, tends to prevent useless variations, and so contributes to the stability of the species. This observer is of opinion that variations are undeniably due to an inborn tendency to vary, which is inherent in the germ plasma of every species of plant and animal, and which is thus an important factor in evolution.

As we come to examine observed differences between individuals and their parents, we discern, as Professor Arthur Thomson states, that many structural peculiarities of the body, can be shown by experiment to be definitely related to some alteration or peculiarity in external environment. This brings us to the great question of the *transmissibility of acquired characters*, than which no subject in connection with heredity has given rise to so much controversy.

The question at issue is as to whether the modifications of the body can so specifically affect the reproductive cells that the next generation will inherit, in some measure at least, the altered characters acquired by the parent or parents. Darwin, Hæckel, and Virchow were all strong supporters of the theory of external causes generating effects which are inherited. Weismann on the other hand, at one time denied all inheritance of acquired characters, and thought that the sole fountain of specific change is to be found in the intermingled nuclear plasma of the sex cells. Weismann himself, however, has since admitted that the environment, and more particularly altered nutritive conditions in the body, may cause variations in the germ-plasma, and so alter its constitution that an organism may be produced which is in some respects different from its parents. If this much is granted, then the transmissibility of acquired characters, including the tendency to diseased states, at once becomes possible and even probable. If nutritive vacillations, as Weismann admits, can produce ever so little modification in the germ plasm, it is easy to conceive that the nuclear plasma cannot remain absolutely unaffected by the physico-chemical conditions of the body (soma).

Let us now consider some of the special acquired conditions which are believed to be hereditary. There is no evidence of the supposed transmission of *mutilations*; the Jews have been circumcised since the days of Abraham, and yet their sons are born with similar foreskins to those of other races. The long continued compression of the feet of Chinese ladies has not resulted in any hereditary change in the feet of Chinese babies. Sir Herman Weber, who has carefully studied the question of *longevity*, is strongly of opinion that heredity exercises a potent influence upon the duration of life. He states that those whose parents and ancestors were long-lived may expect to live longer than the descendants of comparatively short-lived ancestors. The tendency to premature senile degeneration is undoubtedly inherited by certain families. In many of these cases what is actually inherited is a tendency to early vascular degeneration, which may in turn be due to an inherited faulty metabolism. It is a well-known aphorism that a man is as "old as his arteries." Early arterial degeneration predisposes to the occurrence of cardiac degeneration and cerebral hæmorrhage. Dr. Savage mentions the fact that senile mental changes also not infrequently recur in families, and states that he has known a good many instances in which members of certain families died first in their brains.

We will next take up seriatim some of the best-known examples of so-called hereditary diseases, with the view of critically examining their claim to be considered as coming under such a category.

*(To be concluded.)*

